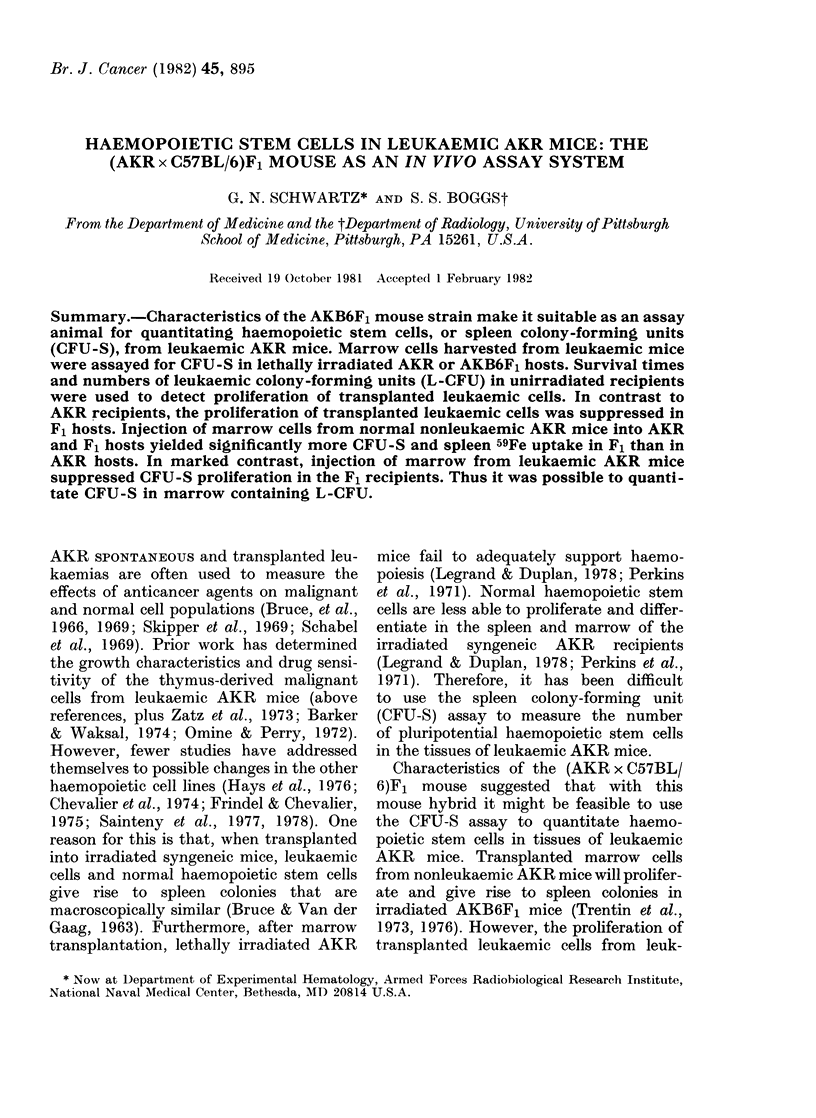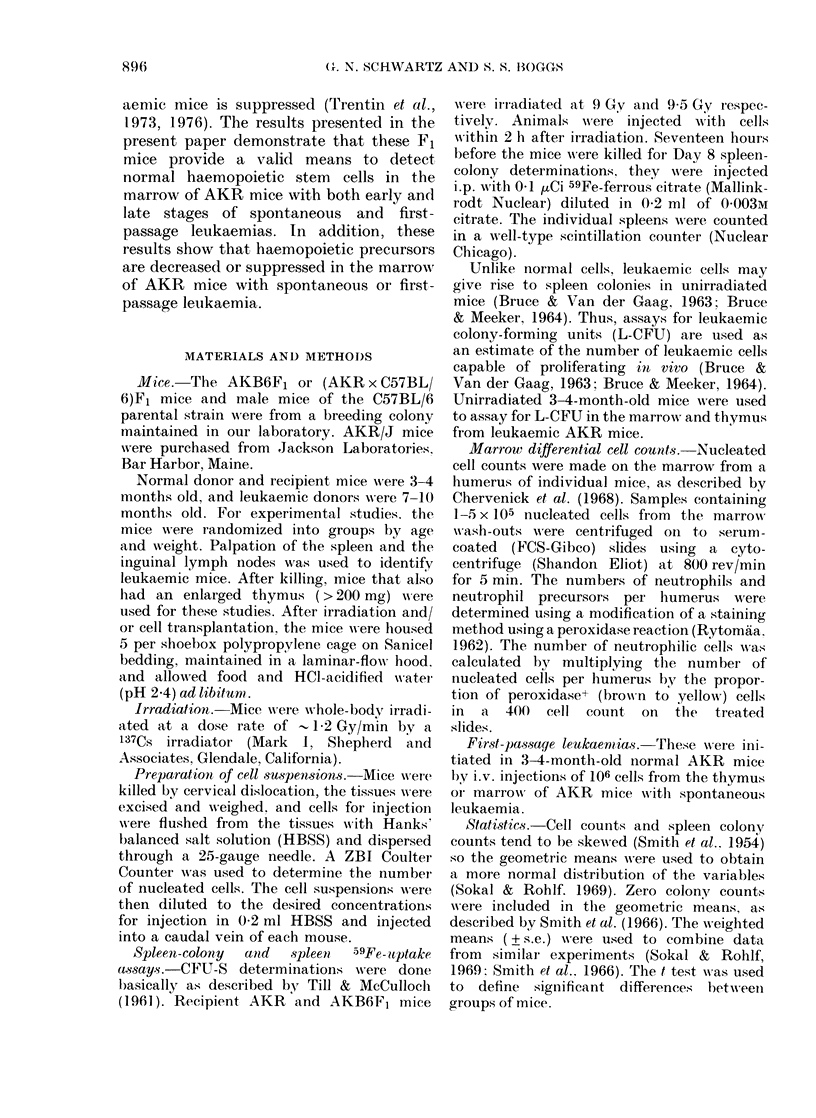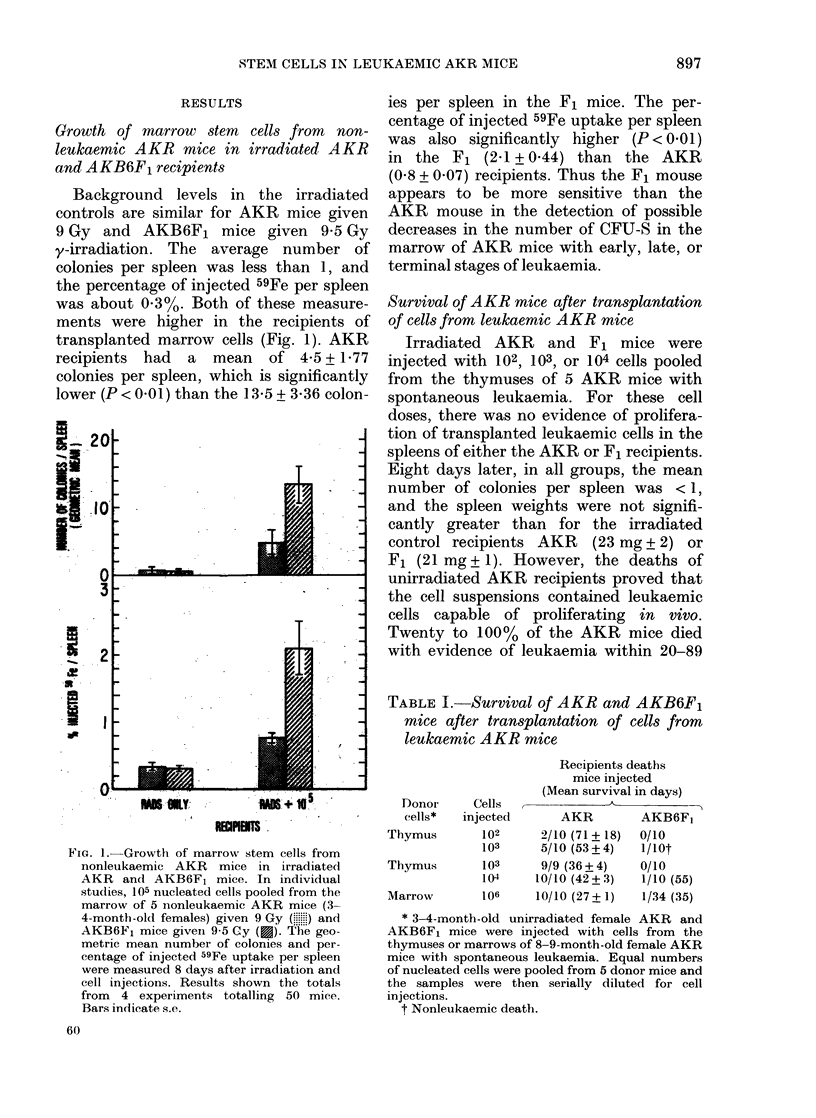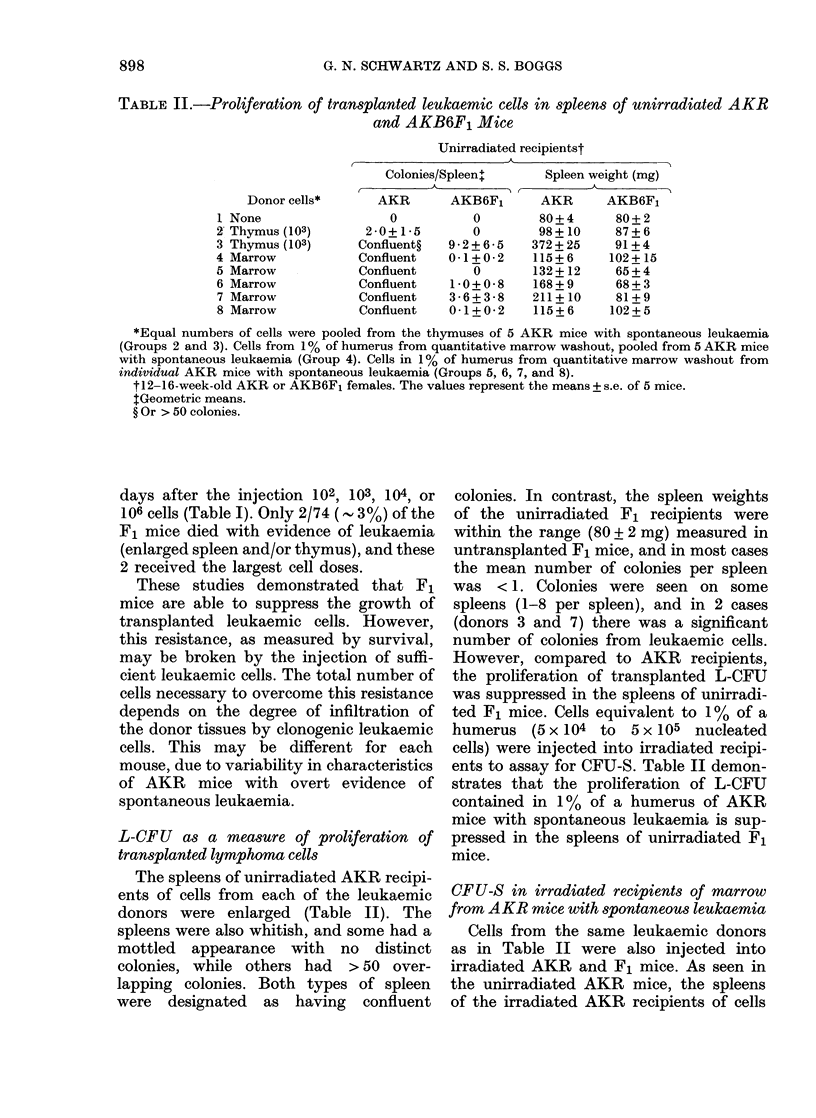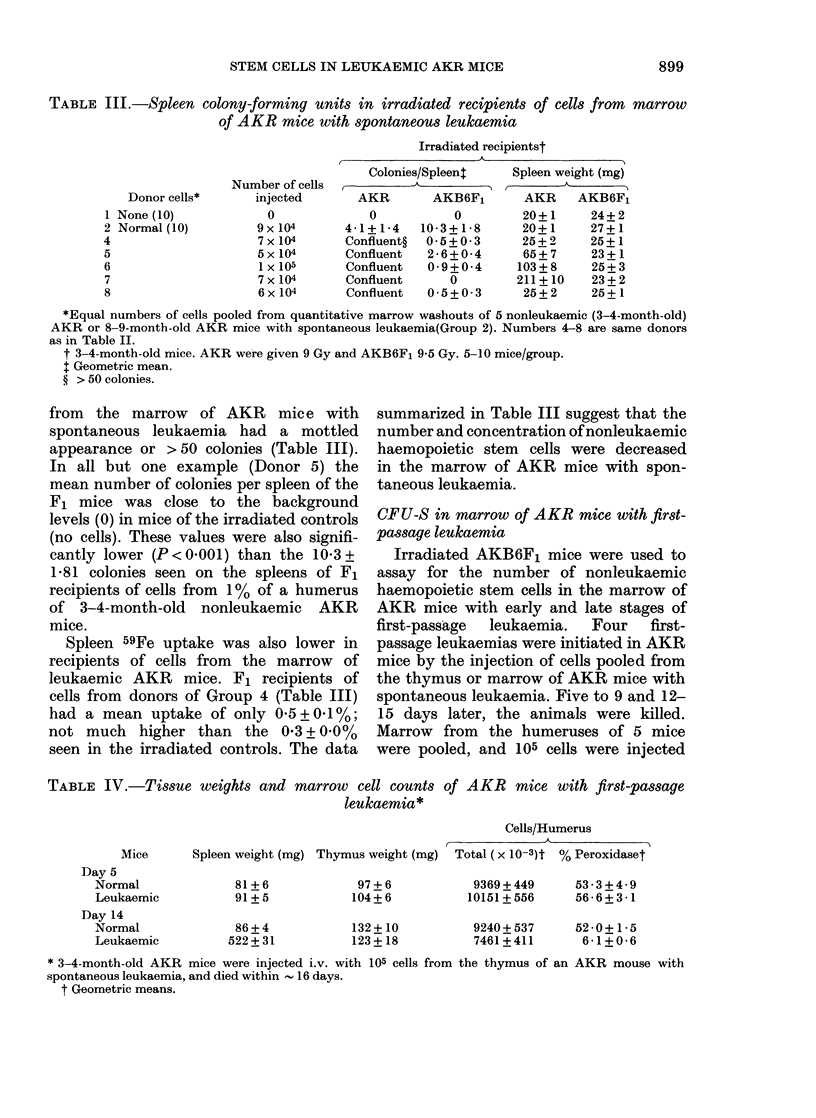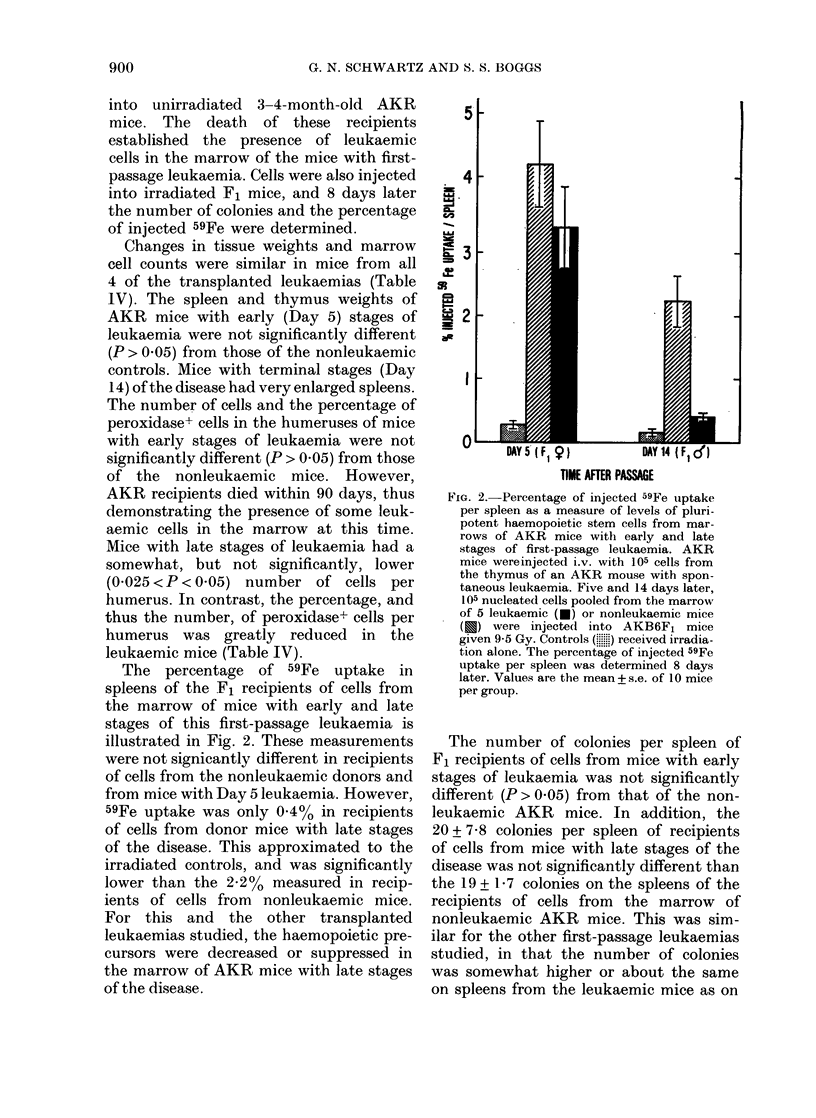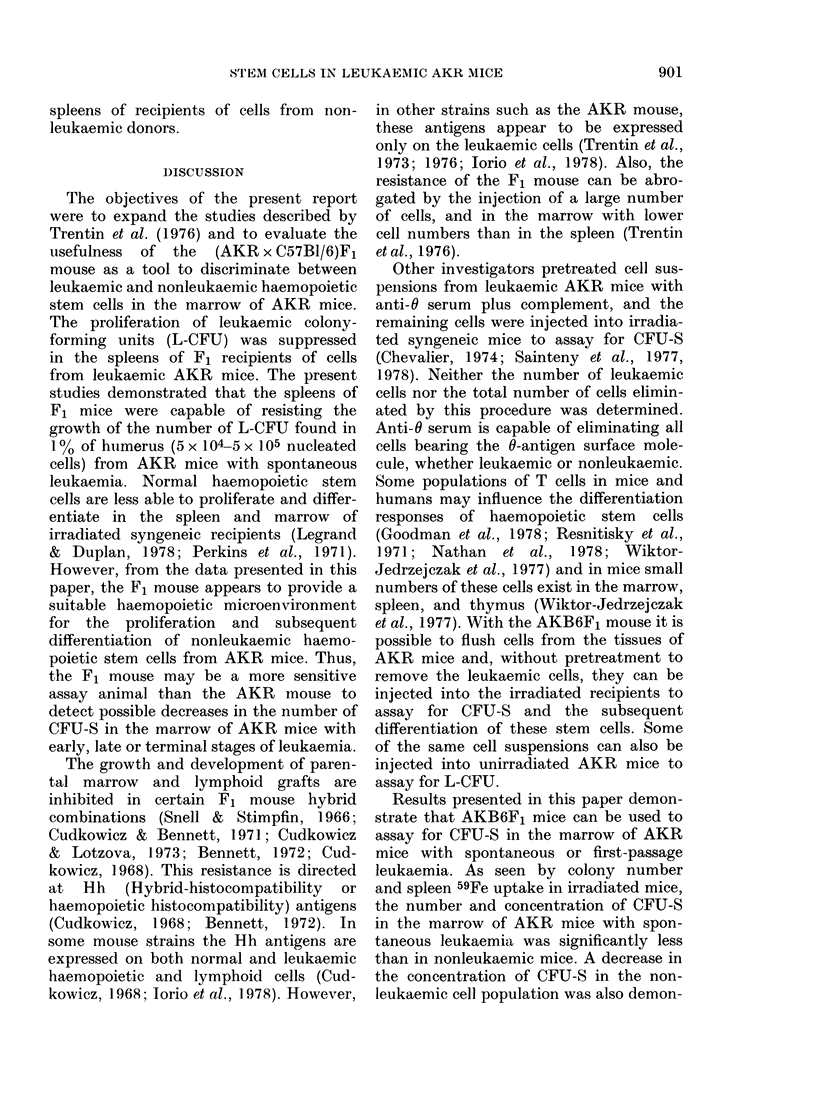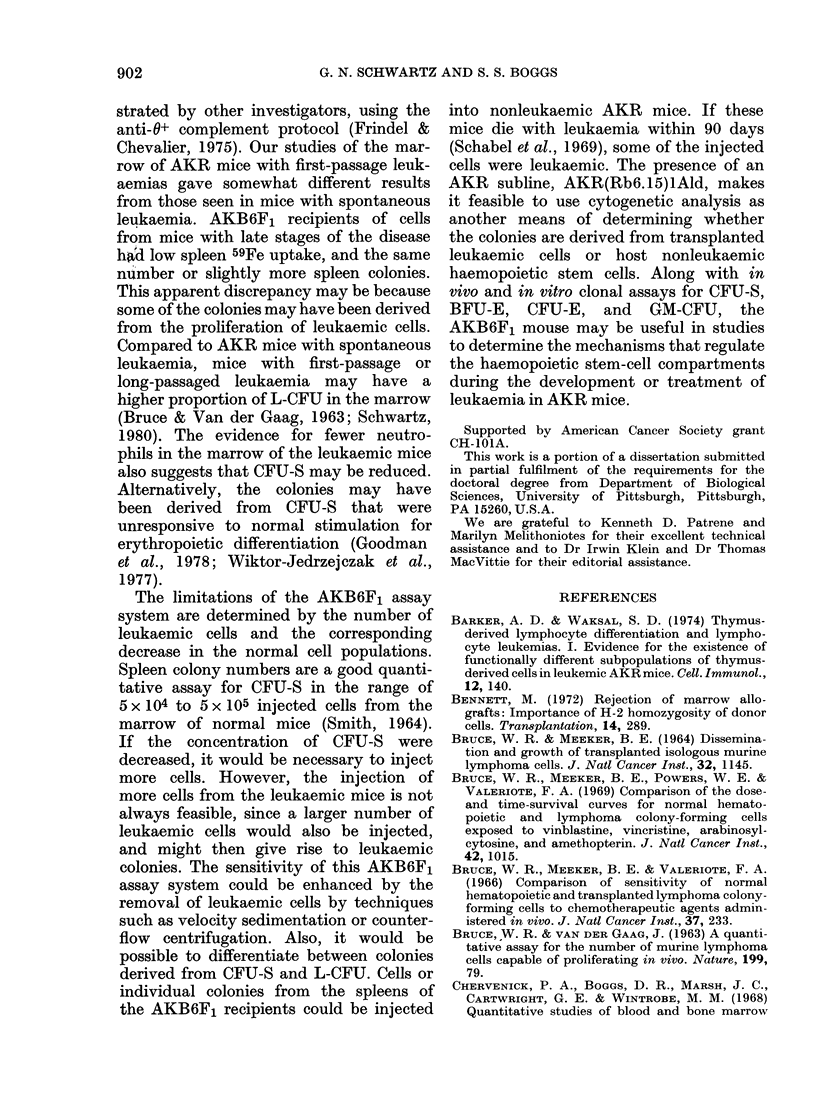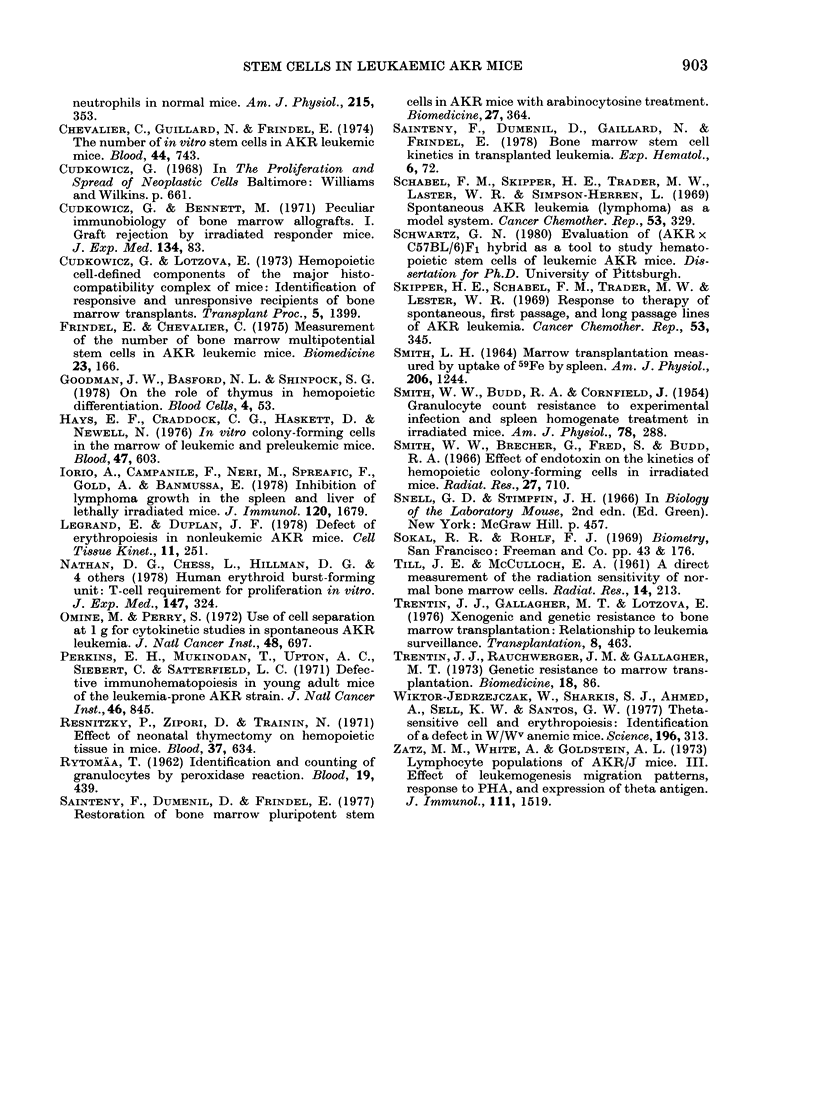# Haemopoietic stem cells in leukaemic AKR mice: the (AKR x C57BL/6)F1 mouse as an in vivo assay system.

**DOI:** 10.1038/bjc.1982.142

**Published:** 1982-06

**Authors:** G. N. Schwartz, S. S. Boggs

## Abstract

Characteristics of the AKB6F1 mouse strain make it suitable as an assay animal for quantitating haemopoietic stem cells, or spleen colony-forming units (CFU-S), from leukaemic AKR mice. Marrow cells harvested from leukaemic mice were assayed for CFU-S in lethally irradiated AKR or AKB6F1 hosts. Survival times and numbers of leukaemic colony-forming units (L-CFU) in unirradiated recipients were used to detect proliferation of transplanted leukaemic cells. In contrast to AKR recipients, the proliferation of transplanted leukaemic cells was suppressed in F1 hosts. Injection of marrow cells from normal nonleukaemic AKR mice into AKR and F1 hosts yielded significantly more CFU-S and spleen 59Fe uptake in F1 than in AKR hosts. In marked contrast, injection of marrow from leukaemic AKR mice suppressed CFU-S proliferation in the F1 recipients. Thus it was possible to quantitate CFU-S in marrow containing L-CFU.


Br. J. Cancer (1982) 45, 895

### HAEMOPOIETIC STEM CELLS IN LEUKAEMIC AKR MICE: THE (AKR × C57BL/6)$F_1$ MOUSE AS AN *IN VIVO* ASSAY SYSTEM

G. N. SCHWARTZ* AND S. S. BOGGS†

*From the Department of Medicine and the †Department of Radiology, University of Pittsburgh School of Medicine, Pittsburgh, PA 15261, U.S.A.*

Received 19 October 1981 Accepted 1 February 1982

**Summary.—Characteristics of the AKB6$F_1$ mouse strain make it suitable as an assay animal for quantitating haemopoietic stem cells, or spleen colony-forming units (CFU-S), from leukaemic AKR mice. Marrow cells harvested from leukaemic mice were assayed for CFU-S in lethally irradiated AKR or AKB6$F_1$ hosts. Survival times and numbers of leukaemic colony-forming units (L-CFU) in unirradiated recipients were used to detect proliferation of transplanted leukaemic cells. In contrast to AKR recipients, the proliferation of transplanted leukaemic cells was suppressed in $F_1$ hosts. Injection of marrow cells from normal nonleukaemic AKR mice into AKR and $F_1$ hosts yielded significantly more CFU-S and spleen $^{59}Fe$ uptake in $F_1$ than in AKR hosts. In marked contrast, injection of marrow from leukaemic AKR mice suppressed CFU-S proliferation in the $F_1$ recipients. Thus it was possible to quantitate CFU-S in marrow containing L-CFU.**

AKR SPONTANEOUS and transplanted leukaemias are often used to measure the effects of anticancer agents on malignant and normal cell populations (Bruce, *et al.*, 1966, 1969; Skipper *et al.*, 1969; Schabel *et al.*, 1969). Prior work has determined the growth characteristics and drug sensitivity of the thymus-derived malignant cells from leukaemic AKR mice (above references, plus Zatz *et al.*, 1973; Barker & Waksal, 1974; Omine & Perry, 1972). However, fewer studies have addressed themselves to possible changes in the other haemopoietic cell lines (Hays *et al.*, 1976; Chevalier *et al.*, 1974; Frindel & Chevalier, 1975; Sainteny *et al.*, 1977, 1978). One reason for this is that, when transplanted into irradiated syngeneic mice, leukaemic cells and normal haemopoietic stem cells give rise to spleen colonies that are macroscopically similar (Bruce & Van der Gaag, 1963). Furthermore, after marrow transplantation, lethally irradiated AKR mice fail to adequately support haemopoiesis (Legrand & Duplan, 1978; Perkins *et al.*, 1971). Normal haemopoietic stem cells are less able to proliferate and differentiate in the spleen and marrow of the irradiated syngeneic AKR recipients (Legrand & Duplan, 1978; Perkins *et al.*, 1971). Therefore, it has been difficult to use the spleen colony-forming unit (CFU-S) assay to measure the number of pluripotential haemopoietic stem cells in the tissues of leukaemic AKR mice.

Characteristics of the (AKR × C57BL/6)$F_1$ mouse suggested that with this mouse hybrid it might be feasible to use the CFU-S assay to quantitate haemopoietic stem cells in tissues of leukaemic AKR mice. Transplanted marrow cells from nonleukaemic AKR mice will proliferate and give rise to spleen colonies in irradiated AKB6$F_1$ mice (Trentin *et al.*, 1973, 1976). However, the proliferation of transplanted leukaemic cells from leuk-

* Now at Department of Experimental Hematology, Armed Forces Radiobiological Research Institute, National Naval Medical Center, Bethesda, MD 20814 U.S.A.

aemic mice is suppressed (Trentin *et al.*, 1973, 1976). The results presented in the present paper demonstrate that these $F_1$ mice provide a valid means to detect normal haemopoietic stem cells in the marrow of AKR mice with both early and late stages of spontaneous and first-passage leukaemias. In addition, these results show that haemopoietic precursors are decreased or suppressed in the marrow of AKR mice with spontaneous or first-passage leukaemia.

### MATERIALS AND METHODS

*Mice.*—The AKB6$F_1$ or (AKR × C57BL/6)$F_1$ mice and male mice of the C57BL/6 parental strain were from a breeding colony maintained in our laboratory. AKR/J mice were purchased from Jackson Laboratories, Bar Harbor, Maine.

Normal donor and recipient mice were 3–4 months old, and leukaemic donors were 7–10 months old. For experimental studies, the mice were randomized into groups by age and weight. Palpation of the spleen and the inguinal lymph nodes was used to identify leukaemic mice. After killing, mice that also had an enlarged thymus ($>200$ mg) were used for these studies. After irradiation and/or cell transplantation, the mice were housed 5 per shoebox polypropylene cage on Sanicel bedding, maintained in a laminar-flow hood, and allowed food and HCl-acidified water (pH 2·4) *ad libitum*.

*Irradiation.*—Mice were whole-body irradiated at a dose rate of ~1·2 Gy/min by a $^{137}Cs$ irradiator (Mark I, Shepherd and Associates, Glendale, California).

*Preparation of cell suspensions.*—Mice were killed by cervical dislocation, the tissues were excised and weighed, and cells for injection were flushed from the tissues with Hanks' balanced salt solution (HBSS) and dispersed through a 25-gauge needle. A ZBI Coulter Counter was used to determine the number of nucleated cells. The cell suspensions were then diluted to the desired concentrations for injection in 0·2 ml HBSS and injected into a caudal vein of each mouse.

*Spleen-colony and spleen $^{59}$Fe-uptake assays.*—CFU-S determinations were done basically as described by Till & McCulloch (1961). Recipient AKR and AKB6$F_1$ mice were irradiated at 9 Gy and 9·5 Gy respectively. Animals were injected with cells within 2 h after irradiation. Seventeen hours before the mice were killed for Day 8 spleen-colony determinations, they were injected i.p. with 0·1 $\mu$Ci $^{59}$Fe-ferrous citrate (Mallinkrodt Nuclear) diluted in 0·2 ml of 0·003M citrate. The individual spleens were counted in a well-type scintillation counter (Nuclear Chicago).

Unlike normal cells, leukaemic cells may give rise to spleen colonies in unirradiated mice (Bruce & Van der Gaag, 1963; Bruce & Meeker, 1964). Thus, assays for leukaemic colony-forming units (L-CFU) are used as an estimate of the number of leukaemic cells capable of proliferating *in vivo* (Bruce & Van der Gaag, 1963; Bruce & Meeker, 1964). Unirradiated 3–4-month-old mice were used to assay for L-CFU in the marrow and thymus from leukaemic AKR mice.

*Marrow differential cell counts.*—Nucleated cell counts were made on the marrow from a humerus of individual mice, as described by Chervenick *et al.* (1968). Samples containing $1–5 \times 10^5$ nucleated cells from the marrow wash-outs were centrifuged on to serum-coated (FCS-Gibco) slides using a cytocentrifuge (Shandon Eliot) at 800 rev/min for 5 min. The numbers of neutrophils and neutrophil precursors per humerus were determined using a modification of a staining method using a peroxidase reaction (Rytomäa, 1962). The number of neutrophilic cells was calculated by multiplying the number of nucleated cells per humerus by the proportion of peroxidase$^+$ (brown to yellow) cells in a 400 cell count on the treated slides.

*First-passage leukaemias.*—These were initiated in 3–4-month-old normal AKR mice by i.v. injections of $10^6$ cells from the thymus or marrow of AKR mice with spontaneous leukaemia.

*Statistics.*—Cell counts and spleen colony counts tend to be skewed (Smith *et al.*, 1954) so the geometric means were used to obtain a more normal distribution of the variables (Sokal & Rohlf, 1969). Zero colony counts were included in the geometric means, as described by Smith *et al.* (1966). The weighted means ($\pm$s.e.) were used to combine data from similar experiments (Sokal & Rohlf, 1969; Smith *et al.*, 1966). The *t* test was used to define significant differences between groups of mice.

### RESULTS

### *Growth of marrow stem cells from non-leukaemic AKR mice in irradiated AKR and AKB6F$_1$ recipients*

Background levels in the irradiated controls are similar for AKR mice given 9 Gy and AKB6F$_1$ mice given 9·5 Gy $\gamma$-irradiation. The average number of colonies per spleen was less than 1, and the percentage of injected $^{59}$Fe per spleen was about 0·3%. Both of these measurements were higher in the recipients of transplanted marrow cells (Fig. 1). AKR recipients had a mean of $4{\cdot}5 \pm 1{\cdot}77$ colonies per spleen, which is significantly lower ($P < 0{\cdot}01$) than the $13{\cdot}5 \pm 3{\cdot}36$ colonies per spleen in the F$_1$ mice. The percentage of injected $^{59}$Fe uptake per spleen was also significantly higher ($P < 0{\cdot}01$) in the F$_1$ ($2{\cdot}1 \pm 0{\cdot}44$) than the AKR ($0{\cdot}8 \pm 0{\cdot}07$) recipients. Thus the F$_1$ mouse appears to be more sensitive than the AKR mouse in the detection of possible decreases in the number of CFU-S in the marrow of AKR mice with early, late, or terminal stages of leukaemia.

FIG. 1.—Growth of marrow stem cells from nonleukaemic AKR mice in irradiated AKR and AKB6F$_1$ mice. In individual studies, $10^5$ nucleated cells pooled from the marrow of 5 nonleukaemic AKR mice (3–4-month-old females) given 9 Gy (dotted) and AKB6F$_1$ mice given 9·5 Gy (hatched). The geometric mean number of colonies and percentage of injected $^{59}$Fe uptake per spleen were measured 8 days after irradiation and cell injections. Results shown the totals from 4 experiments totalling 50 mice. Bars indicate s.e.

### *Survival of AKR mice after transplantation of cells from leukaemic AKR mice*

Irradiated AKR and F$_1$ mice were injected with $10^2$, $10^3$, or $10^4$ cells pooled from the thymuses of 5 AKR mice with spontaneous leukaemia. For these cell doses, there was no evidence of proliferation of transplanted leukaemic cells in the spleens of either the AKR or F$_1$ recipients. Eight days later, in all groups, the mean number of colonies per spleen was $< 1$, and the spleen weights were not significantly greater than for the irradiated control recipients AKR (23 mg $\pm$ 2) or F$_1$ (21 mg $\pm$ 1). However, the deaths of unirradiated AKR recipients proved that the cell suspensions contained leukaemic cells capable of proliferating *in vivo*. Twenty to 100% of the AKR mice died with evidence of leukaemia within 20–89

TABLE I.—*Survival of AKR and AKB6F$_1$ mice after transplantation of cells from leukaemic AKR mice*

| Donor cells* | Cells injected | Recipients deaths / mice injected (Mean survival in days) AKR | AKB6F$_1$ |
|---|---|---|---|
| Thymus | $10^2$ | 2/10 (71 ± 18) | 0/10 |
| | $10^3$ | 5/10 (53 ± 4) | 1/10† |
| Thymus | $10^3$ | 9/9 (36 ± 4) | 0/10 |
| | $10^4$ | 10/10 (42 ± 3) | 1/10 (55) |
| Marrow | $10^6$ | 10/10 (27 ± 1) | 1/34 (35) |

* 3–4-month-old unirradiated female AKR and AKB6F$_1$ mice were injected with cells from the thymuses or marrows of 8–9-month-old female AKR mice with spontaneous leukaemia. Equal numbers of nucleated cells were pooled from 5 donor mice and the samples were then serially diluted for cell injections.

† Nonleukaemic death.

TABLE II.—*Proliferation of transplanted leukaemic cells in spleens of unirradiated AKR and AKB6F₁ Mice*

| | Unirradiated recipients† | | | |
|---|---|---|---|---|
| | Colonies/Spleen‡ | | Spleen weight (mg) | |
| Donor cells* | AKR | AKB6$F_1$ | AKR | AKB6$F_1$ |
| 1 None | 0 | 0 | $80 \pm 4$ | $80 \pm 2$ |
| 2 Thymus ($10^3$) | $2 \cdot 0 \pm 1 \cdot 5$ | 0 | $98 \pm 10$ | $87 \pm 6$ |
| 3 Thymus ($10^3$) | Confluent§ | $9 \cdot 2 \pm 6 \cdot 5$ | $372 \pm 25$ | $91 \pm 4$ |
| 4 Marrow | Confluent | $0 \cdot 1 \pm 0 \cdot 2$ | $115 \pm 6$ | $102 \pm 15$ |
| 5 Marrow | Confluent | 0 | $132 \pm 12$ | $65 \pm 4$ |
| 6 Marrow | Confluent | $1 \cdot 0 \pm 0 \cdot 8$ | $168 \pm 9$ | $68 \pm 3$ |
| 7 Marrow | Confluent | $3 \cdot 6 \pm 3 \cdot 8$ | $211 \pm 10$ | $81 \pm 9$ |
| 8 Marrow | Confluent | $0 \cdot 1 \pm 0 \cdot 2$ | $115 \pm 6$ | $102 \pm 5$ |

*Equal numbers of cells were pooled from the thymuses of 5 AKR mice with spontaneous leukaemia (Groups 2 and 3). Cells from 1% of humerus from quantitative marrow washout, pooled from 5 AKR mice with spontaneous leukaemia (Group 4). Cells in 1% of humerus from quantitative marrow washout from *individual* AKR mice with spontaneous leukaemia (Groups 5, 6, 7, and 8).

†12–16-week-old AKR or AKB6$F_1$ females. The values represent the means ± s.e. of 5 mice.

‡Geometric means.

§Or > 50 colonies.

days after the injection $10^2$, $10^3$, $10^4$, or $10^6$ cells (Table I). Only 2/74 (~3%) of the $F_1$ mice died with evidence of leukaemia (enlarged spleen and/or thymus), and these 2 received the largest cell doses.

These studies demonstrated that $F_1$ mice are able to suppress the growth of transplanted leukaemic cells. However, this resistance, as measured by survival, may be broken by the injection of sufficient leukaemic cells. The total number of cells necessary to overcome this resistance depends on the degree of infiltration of the donor tissues by clonogenic leukaemic cells. This may be different for each mouse, due to variability in characteristics of AKR mice with overt evidence of spontaneous leukaemia.

*L-CFU as a measure of proliferation of transplanted lymphoma cells*

The spleens of unirradiated AKR recipients of cells from each of the leukaemic donors were enlarged (Table II). The spleens were also whitish, and some had a mottled appearance with no distinct colonies, while others had > 50 overlapping colonies. Both types of spleen were designated as having confluent colonies. In contrast, the spleen weights of the unirradiated $F_1$ recipients were within the range ($80 \pm 2$ mg) measured in untransplanted $F_1$ mice, and in most cases the mean number of colonies per spleen was < 1. Colonies were seen on some spleens (1–8 per spleen), and in 2 cases (donors 3 and 7) there was a significant number of colonies from leukaemic cells. However, compared to AKR recipients, the proliferation of transplanted L-CFU was suppressed in the spleens of unirradited $F_1$ mice. Cells equivalent to 1% of a humerus ($5 \times 10^4$ to $5 \times 10^5$ nucleated cells) were injected into irradiated recipients to assay for CFU-S. Table II demonstrates that the proliferation of L-CFU contained in 1% of a humerus of AKR mice with spontaneous leukaemia is suppressed in the spleens of unirradiated $F_1$ mice.

*CFU-S in irradiated recipients of marrow from AKR mice with spontaneous leukaemia*

Cells from the same leukaemic donors as in Table II were also injected into irradiated AKR and $F_1$ mice. As seen in the unirradiated AKR mice, the spleens of the irradiated AKR recipients of cells

TABLE III.—*Spleen colony-forming units in irradiated recipients of cells from marrow of AKR mice with spontaneous leukaemia*

| | | Irradiated recipients† | | | |
|---|---|---|---|---|---|
| | | Colonies/Spleen‡ | | Spleen weight (mg) | |
| Donor cells* | Number of cells injected | AKR | $AKB6F_1$ | AKR | $AKB6F_1$ |
| 1 None (10) | 0 | 0 | 0 | $20\pm1$ | $24\pm2$ |
| 2 Normal (10) | $9\times10^4$ | $4{\cdot}1\pm1{\cdot}4$ | $10{\cdot}3\pm1{\cdot}8$ | $20\pm1$ | $27\pm1$ |
| 4 | $7\times10^4$ | Confluent§ | $0{\cdot}5\pm0{\cdot}3$ | $25\pm2$ | $25\pm1$ |
| 5 | $5\times10^4$ | Confluent | $2{\cdot}6\pm0{\cdot}4$ | $65\pm7$ | $23\pm1$ |
| 6 | $1\times10^5$ | Confluent | $0{\cdot}9\pm0{\cdot}4$ | $103\pm8$ | $25\pm3$ |
| 7 | $7\times10^4$ | Confluent | 0 | $211\pm10$ | $23\pm2$ |
| 8 | $6\times10^4$ | Confluent | $0{\cdot}5\pm0{\cdot}3$ | $25\pm2$ | $25\pm1$ |

*Equal numbers of cells pooled from quantitative marrow washouts of 5 nonleukaemic (3–4-month-old) AKR or 8–9-month-old AKR mice with spontaneous leukaemia(Group 2). Numbers 4–8 are same donors as in Table II.

† 3–4-month-old mice. AKR were given 9 Gy and $AKB6F_1$ 9·5 Gy. 5–10 mice/group.

‡ Geometric mean.

§ > 50 colonies.

from the marrow of AKR mice with spontaneous leukaemia had a mottled appearance or >50 colonies (Table III). In all but one example (Donor 5) the mean number of colonies per spleen of the $F_1$ mice was close to the background levels (0) in mice of the irradiated controls (no cells). These values were also significantly lower ($P < 0{\cdot}001$) than the $10{\cdot}3 \pm 1{\cdot}81$ colonies seen on the spleens of $F_1$ recipients of cells from 1% of a humerus of 3–4-month-old nonleukaemic AKR mice.

Spleen $^{59}Fe$ uptake was also lower in recipients of cells from the marrow of leukaemic AKR mice. $F_1$ recipients of cells from donors of Group 4 (Table III) had a mean uptake of only $0{\cdot}5\pm0{\cdot}1\%$; not much higher than the $0{\cdot}3\pm0{\cdot}0\%$ seen in the irradiated controls. The data summarized in Table III suggest that the number and concentration of nonleukaemic haemopoietic stem cells were decreased in the marrow of AKR mice with spontaneous leukaemia.

### *CFU-S in marrow of AKR mice with first-passage leukaemia*

Irradiated $AKB6F_1$ mice were used to assay for the number of nonleukaemic haemopoietic stem cells in the marrow of AKR mice with early and late stages of first-passage leukaemia. Four first-passage leukaemias were initiated in AKR mice by the injection of cells pooled from the thymus or marrow of AKR mice with spontaneous leukaemia. Five to 9 and 12–15 days later, the animals were killed. Marrow from the humeruses of 5 mice were pooled, and $10^5$ cells were injected

TABLE IV.—*Tissue weights and marrow cell counts of AKR mice with first-passage leukaemia**

| | | | Cells/Humerus | |
|---|---|---|---|---|
| Mice | Spleen weight (mg) | Thymus weight (mg) | Total ($\times10^{-3}$)† | % Peroxidase† |
| Day 5 | | | | |
| Normal | $81\pm6$ | $97\pm6$ | $9369\pm449$ | $53{\cdot}3\pm4{\cdot}9$ |
| Leukaemic | $91\pm5$ | $104\pm6$ | $10151\pm556$ | $56{\cdot}6\pm3{\cdot}1$ |
| Day 14 | | | | |
| Normal | $86\pm4$ | $132\pm10$ | $9240\pm537$ | $52{\cdot}0\pm1{\cdot}5$ |
| Leukaemic | $522\pm31$ | $123\pm18$ | $7461\pm411$ | $6{\cdot}1\pm0{\cdot}6$ |

* 3–4-month-old AKR mice were injected i.v. with $10^5$ cells from the thymus of an AKR mouse with spontaneous leukaemia, and died within ~16 days.

† Geometric means.

into unirradiated 3–4-month-old AKR mice. The death of these recipients established the presence of leukaemic cells in the marrow of the mice with first-passage leukaemia. Cells were also injected into irradiated $F_1$ mice, and 8 days later the number of colonies and the percentage of injected $^{59}Fe$ were determined.

Changes in tissue weights and marrow cell counts were similar in mice from all 4 of the transplanted leukaemias (Table IV). The spleen and thymus weights of AKR mice with early (Day 5) stages of leukaemia were not significantly different ($P > 0{\cdot}05$) from those of the nonleukaemic controls. Mice with terminal stages (Day 14) of the disease had very enlarged spleens. The number of cells and the percentage of peroxidase$^+$ cells in the humeruses of mice with early stages of leukaemia were not significantly different ($P > 0{\cdot}05$) from those of the nonleukaemic mice. However, AKR recipients died within 90 days, thus demonstrating the presence of some leukaemic cells in the marrow at this time. Mice with late stages of leukaemia had a somewhat, but not significantly, lower ($0{\cdot}025 < P < 0{\cdot}05$) number of cells per humerus. In contrast, the percentage, and thus the number, of peroxidase$^+$ cells per humerus was greatly reduced in the leukaemic mice (Table IV).

The percentage of $^{59}Fe$ uptake in spleens of the $F_1$ recipients of cells from the marrow of mice with early and late stages of this first-passage leukaemia is illustrated in Fig. 2. These measurements were not signicantly different in recipients of cells from the nonleukaemic donors and from mice with Day 5 leukaemia. However, $^{59}Fe$ uptake was only 0·4% in recipients of cells from donor mice with late stages of the disease. This approximated to the irradiated controls, and was significantly lower than the 2·2% measured in recipients of cells from nonleukaemic mice. For this and the other transplanted leukaemias studied, the haemopoietic precursors were decreased or suppressed in the marrow of AKR mice with late stages of the disease.

FIG. 2.—Percentage of injected $^{59}Fe$ uptake per spleen as a measure of levels of pluripotent haemopoietic stem cells from marrows of AKR mice with early and late stages of first-passage leukaemia. AKR mice wereinjected i.v. with $10^5$ cells from the thymus of an AKR mouse with spontaneous leukaemia. Five and 14 days later, $10^5$ nucleated cells pooled from the marrow of 5 leukaemic (■) or nonleukaemic mice (▨) were injected into AKB6$F_1$ mice given 9·5 Gy. Controls (▦) received irradiation alone. The percentage of injected $^{59}Fe$ uptake per spleen was determined 8 days later. Values are the mean ± s.e. of 10 mice per group.

The number of colonies per spleen of $F_1$ recipients of cells from mice with early stages of leukaemia was not significantly different ($P > 0{\cdot}05$) from that of the nonleukaemic AKR mice. In addition, the $20 \pm 7{\cdot}8$ colonies per spleen of recipients of cells from mice with late stages of the disease was not significantly different than the $19 \pm 1{\cdot}7$ colonies on the spleens of the recipients of cells from the marrow of nonleukaemic AKR mice. This was similar for the other first-passage leukaemias studied, in that the number of colonies was somewhat higher or about the same on spleens from the leukaemic mice as on spleens of recipients of cells from nonleukaemic donors.

### DISCUSSION

The objectives of the present report were to expand the studies described by Trentin *et al.* (1976) and to evaluate the usefulness of the (AKR × C57Bl/6)$F_1$ mouse as a tool to discriminate between leukaemic and nonleukaemic haemopoietic stem cells in the marrow of AKR mice. The proliferation of leukaemic colony-forming units (L-CFU) was suppressed in the spleens of $F_1$ recipients of cells from leukaemic AKR mice. The present studies demonstrated that the spleens of $F_1$ mice were capable of resisting the growth of the number of L-CFU found in 1% of humerus ($5 \times 10^4$–$5 \times 10^5$ nucleated cells) from AKR mice with spontaneous leukaemia. Normal haemopoietic stem cells are less able to proliferate and differentiate in the spleen and marrow of irradiated syngeneic recipients (Legrand & Duplan, 1978; Perkins *et al.*, 1971). However, from the data presented in this paper, the $F_1$ mouse appears to provide a suitable haemopoietic microenvironment for the proliferation and subsequent differentiation of nonleukaemic haemopoietic stem cells from AKR mice. Thus, the $F_1$ mouse may be a more sensitive assay animal than the AKR mouse to detect possible decreases in the number of CFU-S in the marrow of AKR mice with early, late or terminal stages of leukaemia.

The growth and development of parental marrow and lymphoid grafts are inhibited in certain $F_1$ mouse hybrid combinations (Snell & Stimpfin, 1966; Cudkowicz & Bennett, 1971; Cudkowicz & Lotzova, 1973; Bennett, 1972; Cudkowicz, 1968). This resistance is directed at Hh (Hybrid-histocompatibility or haemopoietic histocompatibility) antigens (Cudkowicz, 1968; Bennett, 1972). In some mouse strains the Hh antigens are expressed on both normal and leukaemic haemopoietic and lymphoid cells (Cudkowicz, 1968; Iorio *et al.*, 1978). However, in other strains such as the AKR mouse, these antigens appear to be expressed only on the leukaemic cells (Trentin *et al.*, 1973; 1976; Iorio *et al.*, 1978). Also, the resistance of the $F_1$ mouse can be abrogated by the injection of a large number of cells, and in the marrow with lower cell numbers than in the spleen (Trentin *et al.*, 1976).

Other investigators pretreated cell suspensions from leukaemic AKR mice with anti-$\theta$ serum plus complement, and the remaining cells were injected into irradiated syngeneic mice to assay for CFU-S (Chevalier, 1974; Sainteny *et al.*, 1977, 1978). Neither the number of leukaemic cells nor the total number of cells eliminated by this procedure was determined. Anti-$\theta$ serum is capable of eliminating all cells bearing the $\theta$-antigen surface molecule, whether leukaemic or nonleukaemic. Some populations of T cells in mice and humans may influence the differentiation responses of haemopoietic stem cells (Goodman *et al.*, 1978; Resnitisky *et al.*, 1971; Nathan *et al.*, 1978; Wiktor-Jedrzejczak *et al.*, 1977) and in mice small numbers of these cells exist in the marrow, spleen, and thymus (Wiktor-Jedrzejczak *et al.*, 1977). With the AKB6$F_1$ mouse it is possible to flush cells from the tissues of AKR mice and, without pretreatment to remove the leukaemic cells, they can be injected into the irradiated recipients to assay for CFU-S and the subsequent differentiation of these stem cells. Some of the same cell suspensions can also be injected into unirradiated AKR mice to assay for L-CFU.

Results presented in this paper demonstrate that AKB6$F_1$ mice can be used to assay for CFU-S in the marrow of AKR mice with spontaneous or first-passage leukaemia. As seen by colony number and spleen $^{59}$Fe uptake in irradiated mice, the number and concentration of CFU-S in the marrow of AKR mice with spontaneous leukaemia was significantly less than in nonleukaemic mice. A decrease in the concentration of CFU-S in the nonleukaemic cell population was also demonstrated by other investigators, using the anti-$\theta^+$ complement protocol (Frindel & Chevalier, 1975). Our studies of the marrow of AKR mice with first-passage leukaemias gave somewhat different results from those seen in mice with spontaneous leukaemia. $AKB6F_1$ recipients of cells from mice with late stages of the disease had low spleen $^{59}Fe$ uptake, and the same number or slightly more spleen colonies. This apparent discrepancy may be because some of the colonies may have been derived from the proliferation of leukaemic cells. Compared to AKR mice with spontaneous leukaemia, mice with first-passage or long-passaged leukaemia may have a higher proportion of L-CFU in the marrow (Bruce & Van der Gaag, 1963; Schwartz, 1980). The evidence for fewer neutrophils in the marrow of the leukaemic mice also suggests that CFU-S may be reduced. Alternatively, the colonies may have been derived from CFU-S that were unresponsive to normal stimulation for erythropoietic differentiation (Goodman *et al.*, 1978; Wiktor-Jedrzejczak *et al.*, 1977).

The limitations of the $AKB6F_1$ assay system are determined by the number of leukaemic cells and the corresponding decrease in the normal cell populations. Spleen colony numbers are a good quantitative assay for CFU-S in the range of $5 \times 10^4$ to $5 \times 10^5$ injected cells from the marrow of normal mice (Smith, 1964). If the concentration of CFU-S were decreased, it would be necessary to inject more cells. However, the injection of more cells from the leukaemic mice is not always feasible, since a larger number of leukaemic cells would also be injected, and might then give rise to leukaemic colonies. The sensitivity of this $AKB6F_1$ assay system could be enhanced by the removal of leukaemic cells by techniques such as velocity sedimentation or counterflow centrifugation. Also, it would be possible to differentiate between colonies derived from CFU-S and L-CFU. Cells or individual colonies from the spleens of the $AKB6F_1$ recipients could be injected into nonleukaemic AKR mice. If these mice die with leukaemia within 90 days (Schabel *et al.*, 1969), some of the injected cells were leukaemic. The presence of an AKR subline, AKR(Rb6.15)1Ald, makes it feasible to use cytogenetic analysis as another means of determining whether the colonies are derived from transplanted leukaemic cells or host nonleukaemic haemopoietic stem cells. Along with *in vivo* and *in vitro* clonal assays for CFU-S, BFU-E, CFU-E, and GM-CFU, the $AKB6F_1$ mouse may be useful in studies to determine the mechanisms that regulate the haemopoietic stem-cell compartments during the development or treatment of leukaemia in AKR mice.

Supported by American Cancer Society grant CH-101A.

This work is a portion of a dissertation submitted in partial fulfilment of the requirements for the doctoral degree from Department of Biological Sciences, University of Pittsburgh, Pittsburgh, PA 15260, U.S.A.

We are grateful to Kenneth D. Patrene and Marilyn Melithoniotes for their excellent technical assistance and to Dr Irwin Klein and Dr Thomas MacVittie for their editorial assistance.

### REFERENCES

BARKER, A. D. & WAKSAL, S. D. (1974) Thymus-derived lymphocyte differentiation and lymphocyte leukemias. I. Evidence for the existence of functionally different subpopulations of thymus-derived cells in leukemic AKR mice. *Cell. Immunol.*, **12**, 140.

BENNETT, M. (1972) Rejection of marrow allografts: Importance of H-2 homozygosity of donor cells. *Transplantation*, **14**, 289.

BRUCE, W. R. & MEEKER, B. E. (1964) Dissemination and growth of transplanted isologous murine lymphoma cells. *J. Natl Cancer Inst.*, **32**, 1145.

BRUCE, W. R., MEEKER, B. E., POWERS, W. E. & VALERIOTE, F. A. (1969) Comparison of the dose- and time-survival curves for normal hematopoietic and lymphoma colony-forming cells exposed to vinblastine, vincristine, arabinosylcytosine, and amethopterin. *J. Natl Cancer Inst.*, **42**, 1015.

BRUCE, W. R., MEEKER, B. E. & VALERIOTE, F. A. (1966) Comparison of sensitivity of normal hematopoietic and transplanted lymphoma colony-forming cells to chemotherapeutic agents administered *in vivo*. *J. Natl Cancer Inst.*, **37**, 233.

BRUCE, W. R. & VAN DER GAAG, J. (1963) A quantitative assay for the number of murine lymphoma cells capable of proliferating *in vivo*. *Nature*, **199**, 79.

CHERVENICK, P. A., BOGGS, D. R., MARSH, J. C., CARTWRIGHT, G. E. & WINTROBE, M. M. (1968) Quantitative studies of blood and bone marrow neutrophils in normal mice. *Am. J. Physiol.*, **215**, 353.

CHEVALIER, C., GUILLARD, N. & FRINDEL, E. (1974) The number of *in vitro* stem cells in AKR leukemic mice. *Blood*, **44**, 743.

CUDKOWICZ, G. (1968) In *The Proliferation and Spread of Neoplastic Cells* Baltimore: Williams and Wilkins. p. 661.

CUDKOWICZ, G. & BENNETT, M. (1971) Peculiar immunobiology of bone marrow allografts. I. Graft rejection by irradiated responder mice. *J. Exp. Med.* **134**, 83.

CUDKOWICZ, G. & LOTZOVA, E. (1973) Hemopoietic cell-defined components of the major histocompatibility complex of mice: Identification of responsive and unresponsive recipients of bone marrow transplants. *Transplant Proc.*, **5**, 1399.

FRINDEL, E. & CHEVALIER, C. (1975) Measurement of the number of bone marrow multipotential stem cells in AKR leukemic mice. *Biomedicine* **23**, 166.

GOODMAN, J. W., BASFORD, N. L. & SHINPOCK, S. G. (1978) On the role of thymus in hemopoietic differentiation. *Blood Cells*, **4**, 53.

HAYS, E. F., CRADDOCK, C. G., HASKETT, D. & NEWELL, N. (1976) *In vitro* colony-forming cells in the marrow of leukemic and preleukemic mice. *Blood*, **47**, 603.

IORIO, A., CAMPANILE, F., NERI, M., SPREAFIC, F., GOLD, A. & BANMUSSA, E. (1978) Inhibition of lymphoma growth in the spleen and liver of lethally irradiated mice. *J. Immunol.* **120**, 1679.

LEGRAND, E. & DUPLAN, J. F. (1978) Defect of erythropoiesis in nonleukemic AKR mice. *Cell Tissue Kinet.*, **11**, 251.

NATHAN, D. G., CHESS, L., HILLMAN, D. G. & 4 others (1978) Human erythroid burst-forming unit: T-cell requirement for proliferation *in vitro*. *J. Exp. Med.*, **147**, 324.

OMINE, M. & PERRY, S. (1972) Use of cell separation at 1 g for cytokinetic studies in spontaneous AKR leukemia. *J. Natl Cancer Inst.*, **48**, 697.

PERKINS, E. H., MUKINODAN, T., UPTON, A. C., SIEBERT, C. & SATTERFIELD, L. C. (1971) Defective immunohematopoiesis in young adult mice of the leukemia-prone AKR strain. *J. Natl Cancer Inst.*, **46**, 845.

RESNITZKY, P., ZIPORI, D. & TRAININ, N. (1971) Effect of neonatal thymectomy on hemopoietic tissue in mice. *Blood*, **37**, 634.

RYTOMÄA, T. (1962) Identification and counting of granulocytes by peroxidase reaction. *Blood*, **19**, 439.

SAINTENY, F., DUMENIL, D. & FRINDEL, E. (1977) Restoration of bone marrow pluripotent stem cells in AKR mice with arabinocytosine treatment. *Biomedicine*, **27**, 364.

SAINTENY, F., DUMENIL, D., GAILLARD, N. & FRINDEL, E. (1978) Bone marrow stem cell kinetics in transplanted leukemia. *Exp. Hematol.*, **6**, 72.

SCHABEL, F. M., SKIPPER, H. E., TRADER, M. W., LASTER, W. R. & SIMPSON-HERREN, L. (1969) Spontaneous AKR leukemia (lymphoma) as a model system. *Cancer Chemother. Rep.*, **53**, 329.

SCHWARTZ, G. N. (1980) Evaluation of (AKR × C57BL/6)$F_1$ hybrid as a tool to study hematopoietic stem cells of leukemic AKR mice. *Dissertation for Ph.D.* University of Pittsburgh.

SKIPPER, H. E., SCHABEL, F. M., TRADER, M. W. & LESTER, W. R. (1969) Response to therapy of spontaneous, first passage, and long passage lines of AKR leukemia. *Cancer Chemother. Rep.*, **53**, 345.

SMITH, L. H. (1964) Marrow transplantation measured by uptake of $^{59}$Fe by spleen. *Am. J. Physiol.*, **206**, 1244.

SMITH, W. W., BUDD, R. A. & CORNFIELD, J. (1954) Granulocyte count resistance to experimental infection and spleen homogenate treatment in irradiated mice. *Am. J. Physiol.*, **78**, 288.

SMITH, W. W., BRECHER, G., FRED, S. & BUDD, R. A. (1966) Effect of endotoxin on the kinetics of hemopoietic colony-forming cells in irradiated mice. *Radiat. Res.*, **27**, 710.

SNELL, G. D. & STIMPFIN, J. H. (1966) In *Biology of the Laboratory Mouse*, 2nd edn. (Ed. Green). New York: McGraw Hill. p. 457.

SOKAL, R. R. & ROHLF, F. J. (1969) *Biometry*, San Francisco: Freeman and Co. pp. 43 & 176.

TILL, J. E. & MCCULLOCH, E. A. (1961) A direct measurement of the radiation sensitivity of normal bone marrow cells. *Radiat. Res.*, **14**, 213.

TRENTIN, J. J., GALLAGHER, M. T. & LOTZOVA, E. (1976) Xenogenic and genetic resistance to bone marrow transplantation: Relationship to leukemia surveillance. *Transplantation*, **8**, 463.

TRENTIN, J. J., RAUCHWERGER, J. M. & GALLAGHER, M. T. (1973) Genetic resistance to marrow transplantation. *Biomedicine*, **18**, 86.

WIKTOR-JEDRZEJCZAK, W., SHARKIS, S. J., AHMED, A., SELL, K. W. & SANTOS, G. W. (1977) Theta-sensitive cell and erythropoiesis: Identification of a defect in W/$W^v$ anemic mice. *Science*, **196**, 313.

ZATZ, M. M., WHITE, A. & GOLDSTEIN, A. L. (1973) Lymphocyte populations of AKR/J mice. III. Effect of leukemogenesis migration patterns, response to PHA, and expression of theta antigen. *J. Immunol.*, **111**, 1519.